# Co-expression of key gene modules and pathways of human breast cancer cell lines

**DOI:** 10.1042/BSR20181925

**Published:** 2019-07-19

**Authors:** Yadong Wu, Feng liu, Siyang Luo, Xinhai Yin, Dengqi He, Jianguo Liu, Zhaohui Yue, Jukun Song

**Affiliations:** 1 Department of Oral and Maxillofacial Surgery, Guizhou Provincial People’s Hospital, Guizhou, China; 2 Department of Ultrasonic, Guizhou Provincial People’s Hospital, Guizhou, China; 3 Department of Stomatology, First Hospital of Lanzphou University, Lanzhou, China; 4 Special Key Laboratory of Oral Diseases Research, Stomatological Hospital Affiliated to Zunyi Medical University, Guizhou, China

**Keywords:** breast cancer cell lines, GEO, WGCNA

## Abstract

Breast cancer (BC) is the most common leading cause of cancer-related death in women worldwide. Gene expression profiling analysis for human BCs has been studied previously. However, co-expression analysis for BC cell lines is still devoid to date. The aim of the study was to identify key pathways and hub genes that may serve as a biomarker for BC and uncover potential molecular mechanism using weighted correlation network analysis. We analyzed microarray data of BC cell lines (GSE 48213) listed in the Gene Expression Omnibus database. Gene co-expression networks were used to construct and explore the biological function in hub modules using the weighted correlation network analysis algorithm method. Meanwhile, Gene ontology and KEGG pathway analysis were performed using Cytoscape plug-in ClueGo. The network of the key module was also constructed using Cytoscape. A total of 5000 genes were selected, 28 modules of co-expressed genes were identified from the gene co–expression network, one of which was found to be significantly associated with a subtype of BC lines. Functional enrichment analysis revealed that the brown module was mainly involved in the pathway of the autophagy, spliceosome, and mitophagy, the black module was mainly enriched in the pathway of colorectal cancer and pancreatic cancer, and genes in midnightblue module played critical roles in ribosome and regulation of lipolysis in adipocytes pathway. Three hub genes CBR3, SF3B6, and RHPN1 may play an important role in the development and malignancy of the disease. The findings of the present study could improve our understanding of the molecular pathogenesis of breast cancer.

## Introduction

Breast cancer (BC) is one of the most common types of cancer in women [1]. This disease largely affects women in their 40–60s. BC accounts for 29% of all female cancer-related cancer worldwide that contributed the second most cancer now, after lung cancer both in developing and developed countries [2]. At present, the morbidity and mortality of BC are continuously growing. In 2017, approximately 63,990 new cases and 14,440 deaths were predicted to be associated with kidney cancer in the U.S.A. [3]. Nevertheless, BC is a complex disease with respect to molecular alterations, cellular composition, and clinical outcome six subtypes were defined approximately a decade ago based on transcriptional characteristics, and were designated luminal A, luminal B, ERBB2-enriched, basal-like, Claudin-low, and normal-like [4]. However, growing body studies indicated that BC is heterogeneous cancer in various aspects including clinical-pathological, molecular, and cellular heterogeneity [5]. In order to improve the prognosis and decrease mortality and morbidity of BC, diagnostic biomarkers are critical for early detection and risk stratification of BC, which could help us to choose the proper treatment.

Weighted gene co-expression network analysis (WGCNA), a comprehensive collection of R functions, is a prevalent and useful method in the correlation network analysis and the identification of disease-related gene modules and key genes that contribute to phenotypic traits [6,7]. WGCNA approach has provided functional interpretation tools in systems biology. Unlike conventional microarray-based expression profiling method, WGCA allows a global interpretation of gene expression data by constructing gene networks based on similarities in expression profiles amongst samples. To better understand and explore the complex mechanisms of BC cell lines, WGCNA method would be the best choice to study the disease. WGCNA has been successfully performed in various types of cancer, such coronary heart disease [8], uveal melanoma [9], gastric adenocarcinoma [10], prostate cancer [11], and lung cancer [12]. However, the analysis of microarray-based gene expression data by the WGCNA has so far not been applied to compare the co-expression network of BC cell lines.

In the present study, the WGCNA method was applied to analyze a gene expression dataset of the BC cell line to identify biologically relevant modules associated with BC cell line subtypes.

## material and methods

### Expression analysis of microarray data of BC cell line samples

Gene expression profiles of breast cell lines were accessible from the Gene Expression Omnibus (GEO) database using the accession number GSE 48216 [13]. The microarray gene data included 56 BC cell lines, which composed of five subtypes (basal, claudin-low, luminal, non-malignant, and unknown). Annotated files of microarray platform (GPL 10999) were also downloaded from GEO. Annotation information of microarray data was employed to match probes with corresponding gene information. The mean value was used to consider the expression level if one gene detected by multiple probes. We select the top 25% variance gene expression data as our study object. A matrix of pairwise correlations between all pairs of genes across all selected samples was constructed.

### Construction of gene co-expression network analysis

The WGCNA implemented in the R software package (http://www.r-project.org/) is employed to construct the gene co-expression network and identify the co-expression modules. The process is summarized as follows. First, hierarchical clustering analysis was performed on the samples by the flashClust package in R software [14] and found the sample outlier. Second, WGCNA algorithm was used to calculate the correlations amongst genes across samples, and the appropriate soft threshold power was chosen and the standard scale-free network was established by the condition of scale independent as >0.8. Third, module identification was carried out with the dynamic tree cut method by hierarchically clustering the genes using 1- topological overlap matrix (TOM) as the distance measure with a deep split value of 2 and minimum module size (minClusterSize) of 50 for the resulting dendrogram. Highly similar modules were clustered and merged with a height cutoff of 0.25. Finally, WGCNA algorithm was then used to construct the co-expression modules and extract the gene information in each module.

### Establishment of module related trait relationships

The correlation between gene expression modules and each phenotype of BC cell lines was determined by WGCNA. Spearman’s correlation analysis was carried out to determine the association individual module and subtype of BC cell lines, which was the most relevant module between the module eigengene (ME) and clinical traits. Each subtype of BC has a strongly related module, which can be used as its signature. Gene significance (GS) was calculated for each expression profiles, module membership (MM) was defined as the correlation of expression profile and each MM. The GS versus MM plot was also drawn.

### Interaction analysis of co-expression modules of BC

Interaction relationships amongst different co-expression modules were imputed by WGCNA. Heatmap tool package in R software was used in the evaluation of the strength of the relationship. We correlate the clustering coefficient with connectivity by the module in the unweighted network. The heatmap of gene expression profiles in the individual module was also shown.

### Functional enrichment analysis of genes in the hub modules

The number of genes in the critical modules was put in the order from high to low gene significance scores. Then, functional enrichment analysis was conducted on these genes in these modules. GO Biological Process term and Kyoto Encyclopedia of Genes and Genomes (KEGG) pathway analyses were conducted using Cytoscape plug-in ClueGo [15]. Functional enrichment analysis was based on the cut-off value of *P*-value <0.05.

### Module network visualization and hub genes

The interested module was constructed using the Cytoscape software, and Cytoscape plug-in molecular complex detection (MCODE) was used to analyze the most notable clustering module.

### The Kaplan–Meier plotter

The prognostic value of hub gene was evaluated using an online database, Kaplan–Meier plotter (www.kmplot.com) [16], which integrated gene expression data with survival information of 4142 clinical BC patients [17]. For overall survival analysis, the log-rank test was employed to compare the survival difference between the BC group and non-tumor group. A two-sided *P*-value of <0.05 was regarded as statistically significant.

### Validation of the hub gene expression with TCGA and CCLE database

To verify the hub genes from the TCGA database, the expression statuses of hub genes in the TCGA BRCA cohort were revealed for validation. The diagnostic performance was evaluated using receiver operating characteristic (ROC) curves. The hub genes in a different type of cancers and cell lines were determined through analysis in CCLE database (https://portals.broadinstitute.org/ccle/home), which is publicly a large-scale genomic dataset of human cancer cell lines to facilitate discovery and identification from genome-wide expression analyses [18]. *P*-value, less than 0.05, was regarded as significant. All analyses were performed using R/Bioconductor (version 3.3.2).

## Results

### Collection of gene expression data

The raw gene expression data (GSE 48213) were downloaded from the GEO data repository (http://www.ncbi.nlm.nih.gov/geo/). The dataset contained a total of 56 BC cell lines, consisting 27 luminal, 14 basal, six claudin-low, one Kbluc, five non-malignant, and three of unknown subtype. The detailed information is shown in Table 1. The sequencing platform was GPL10999 Illumina Genome Analyzer IIx (*Homo sapiens*). The gene expression matrix was downloaded from the GEO database. The mean value was used to represent the expression level if one gene detected by multiple probes. As a result, a total of 36,953 expression data of genes was obtained. To select the most varying genes, the top highest 25% variance expression profiles were chosen to conduct the WGCNA analysis.

**Table 1:** All samples of BC cell lines

GEO no.	Cell line	Subtype
GSM1172844	184A1	Non-malignant
GSM1172845	184B5	Non-malignant
GSM1172846	21MT1	Basal
GSM1172847	21MT2	Basal
GSM1172848	21NT	Basal
GSM1172849	21PT	Basal
GSM1172850	600MPE	Luminal
GSM1172851	AU565	Luminal
GSM1172853	BT474	Luminal
GSM1172854	BT483	Luminal
GSM1172855	BT549	Claudin-low
GSM1172856	CAMA1	Luminal
GSM1172858	EFM192A	Luminal
GSM1172859	EFM192B	Luminal
GSM1172860	EFM192C	Luminal
GSM1172861	HCC1143	Basal
GSM1172863	HCC1395	Claudin-low
GSM1172864	HCC1419	Luminal
GSM1172865	HCC1428	Luminal
GSM1172867	HCC1569	Basal
GSM1172868	HCC1599	Basal
GSM1172869	HCC1806	Basal
GSM1172870	HCC1937	Basal
GSM1172871	HCC1954	Basal
GSM1172872	HCC202	Luminal
GSM1172873	HCC2218	Luminal
GSM1172874	HCC3153	Basal
GSM1172875	HCC38	Claudin-low
GSM1172876	HCC70	Basal
GSM1172877	HS578T	Claudin-low
GSM1172878	JIMT1	Basal
GSM1172879	LY2	Luminal
GSM1172881	MB157	unknown
GSM1172882	MCF10A	Non-malignant
GSM1172883	MCF10F	Non-malignant
GSM1172884	MCF12A	Non-malignant
GSM1172885	MCF7	Luminal
GSM1172886	MDAMB134VI	Luminal
GSM1172888	MDAMB175	Luminal
GSM1172889	MDAMB231	Claudin-low
GSM1172890	MDAMB361	Luminal
GSM1172893	MX1	unknown
GSM1172895	SKBR3	Luminal
GSM1172896	SUM1315	Claudin-low
GSM1172897	SUM149PT	Basal
GSM1172901	SUM225CWN	Luminal
GSM1172902	SUM229PE	unknown
GSM1172903	SUM52PE	Luminal
GSM1172904	T47D	Luminal
GSM1172905	T47D	Kbluc
GSM1172906	UACC812	Luminal
GSM1172907	UACC893	Luminal
GSM1172908	ZR751	Luminal
GSM1172909	ZR7530	Luminal
GSM1172910	ZR75B	Luminal
GSM1384316	MDAMB453	Luminal

### Construction of co–expression module of BC cell lines

**Figure 1:**
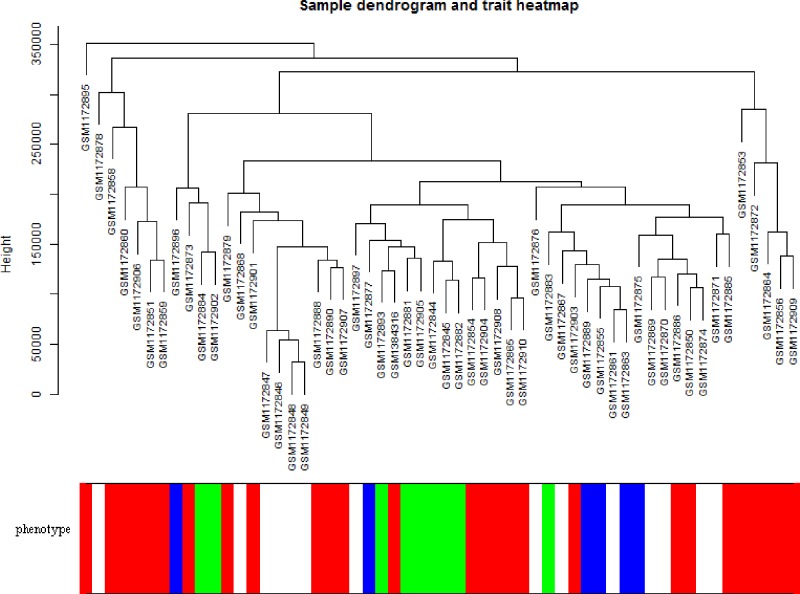
Sample hierarchical clustering plot and trait heatmap Basal represent white, claudin-low represents blue, luminal represents red, and non-malignant represents green.

**Figure 2:**
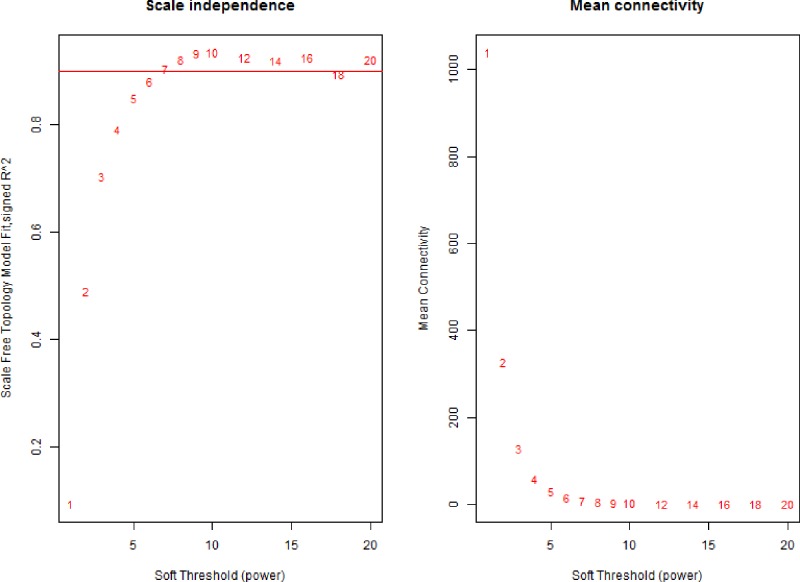
Analysis of network topology for various soft-thresholding powers

**Figure 3:**
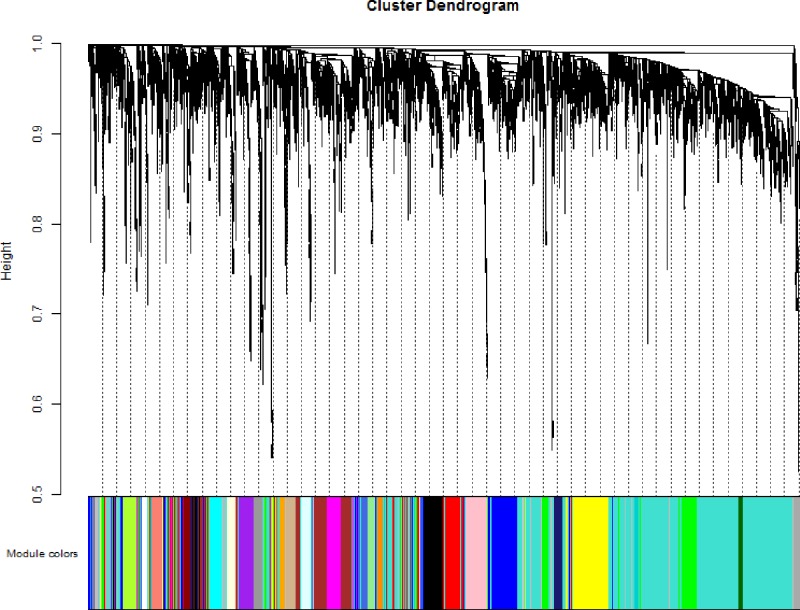
Clustering dendrograms of genes with dissimilarity based on the topological overlap, together with assigned module color

Co-expression modules were constructed by the expression values of 5000 genes in 56 BC cell lines using the WGCNA algorithm. On the whole, the sample hierarchical clustering plot in each sample was divided into two clusters using the flashClust tool package of WGCNA algorithm method. As can be seen in Figure 1, 56 BC samples were divided into two clusters, and we fail to detect outlier samples. Power values were screened out by WGCNA algorithm in the construction of co-expression modules. When the soft threshold power β was set at 6, the independence degree was up to 0.8 and the average connectivity degree was higher (Figure 2). After highly similar modules were merged, a total of 28 co-expression modules were identified in the co-expression network (Figure 3). Total 246 genes in the gray module did not belong to other modules, accounting for 0.492% in all total genes. The number of genes included in these modules was 167 (black module), 355 (blue module), 294 (brown module), 84 (cyan module), 44 (darkgreen module), 40 (darkgrey module), 38 (darkorange module), 43 (darkred module), 41 (darkturquoise), 270 (green module), 101 (greenyellow module), 246 (grey module), 67 (grey60 module), 77 (lightcyan module), 62 (lightgreen module), 61 (lightyellow module), 124 (magenta module), 84 (midnightblue module), 40 (orange module), 168 (pink module), 108 (purple module), 241 (red module), 60 (royalblue module), 87 (salmon module), 88 (tan module), 1671 (turquoise module), 37 (white module), and 279 (yellow module). The average number of genes in these 44 modules was 1671 and the median was 162. The information of genes belonged to four modules was listed in Supplementary Table S1.

### Correlation between modules and each subtype of BC cell lines in co-expression networks

**Figure 4:**
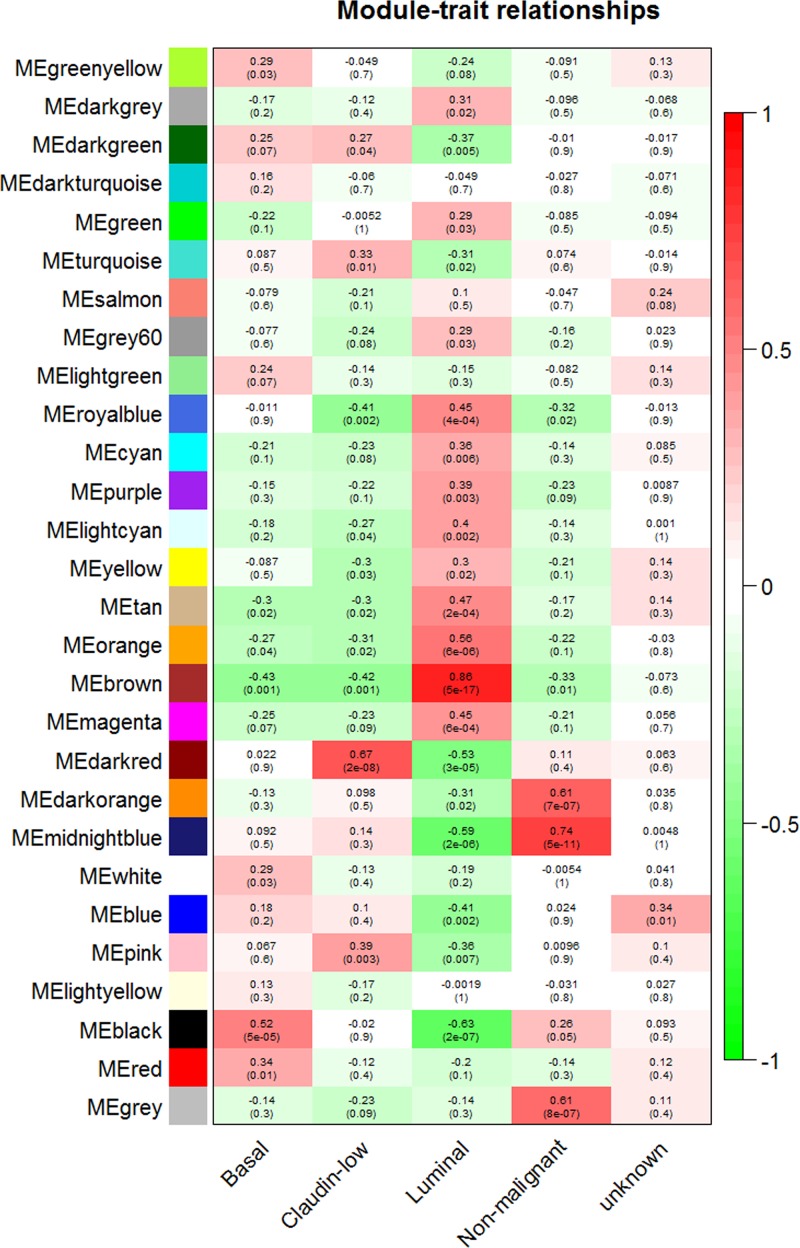
Module-trait associations Each row represents a module eigengene, each column to a trait. Each cell contains the corresponding correlation and *P*-value.

**Figure 5:**
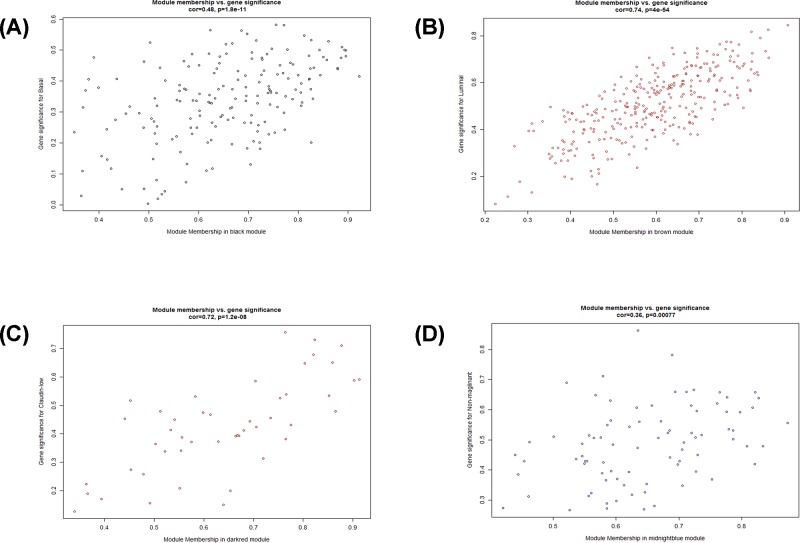
A scatterplot of gene significance versus MM Plot **(A)** is for brown module, plot **(B)** is for the black module, plot **(C)** is for dark red module, and plot **(D)** is for midnight blue module.

The module-trait relationships were established by WGCNA algorithm. Modules with higher Spearman’s correlation coefficients are also the most important elements of modules associated with the trait. From Figure 4, the modules related to BC cell line classification were observed, including the five categories of ‘basal’, ‘claudin-low’, ‘luminal’, ‘non-malignant’, and ‘unknown’. The correlation between ME and traits shows that some modules are more important than others in the disease. A scatterplot of gene significance versus MM was also plotted. We found that the luminal phenotype highly correlated with the brown module (Spearmen correlation coefficients: 0.86, *P*<0.01), with a significantly significant correlation. The basal phenotype is highly related to the black module (Cor: 0.52, *P*<0.01). Claudin-low subtype highly correlated with darkred module (Cor: 0.67, *P*<0.01). Non-malignant subtype was highly associated with midnightblue module (Cor: 0.74, *P*<0.01) (Figure 5). The module with the greatest association with clinical traits was the brown-colored module. Therefore, this module should allow us to capture and summarize the most relevant mRNA expression profiles.

### Interaction analysis of co-expression modules and trait

**Figure 6:**
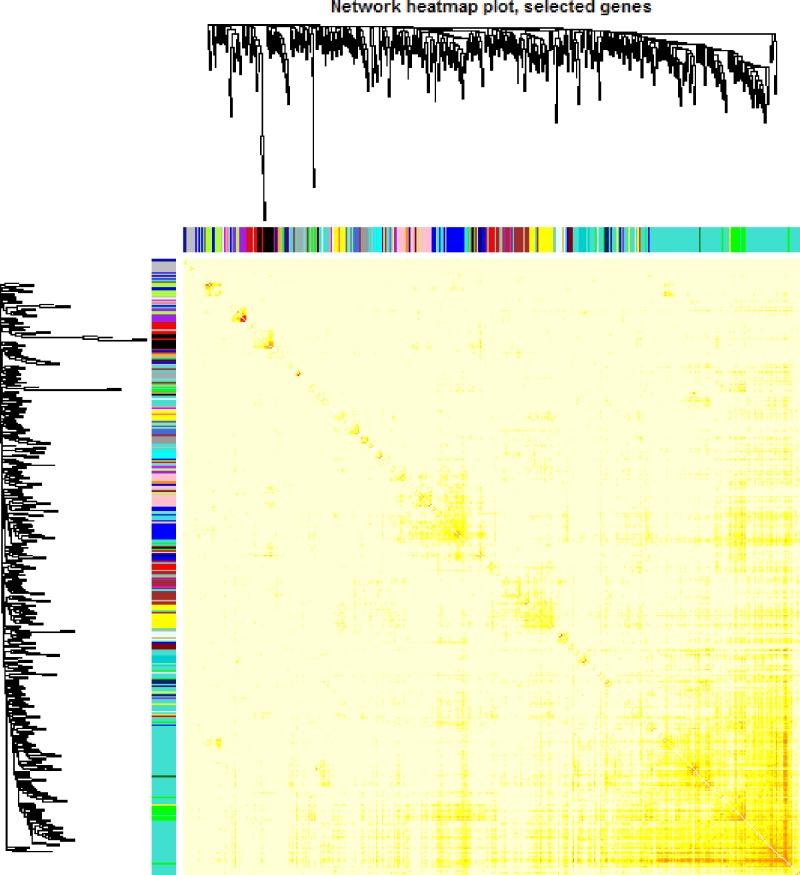
Heat map view of TOM of co-expressed genes in different modules in the top 1500 genes

**Figure 7:**
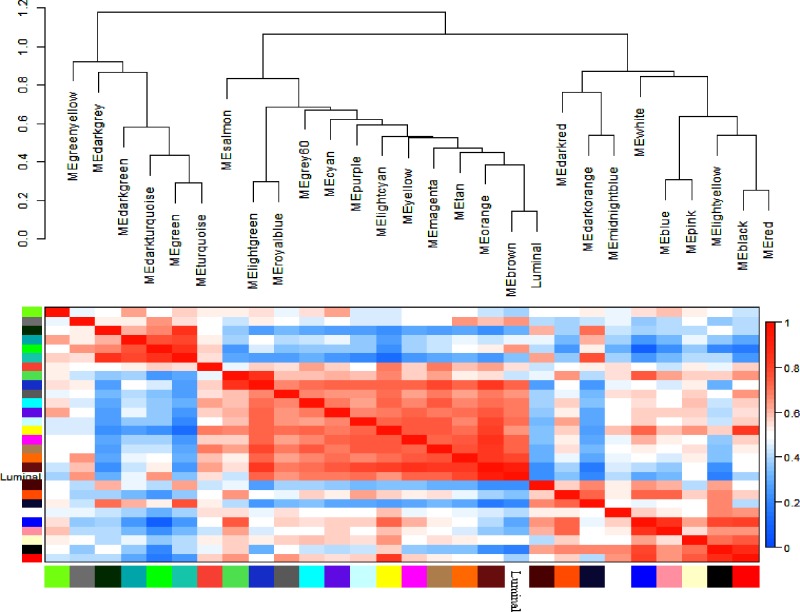
Analysis of connectivity of eigengenes in a different module **(A)** Cluster analysis of eigengenes and **(B)** the heatmap of connectivity of eigengenes.

We further analyzed the interaction amongst 28 co-expression modules. On the whole, no significant difference amongst different modules was observed, suggesting the relative independence of gene expression in these modules (Figure 6). The higher scale independence amongst these modules was also detected. Connectivity of eigengenes analysis was performed in order to evaluate the interactions amongst these constructed co-expression modules (Figure 7). The dendrogram plot also indicated that the brown module was highly associated with luminal BC.

### Functional enrichment analysis of critical modules

**Figure 8:**
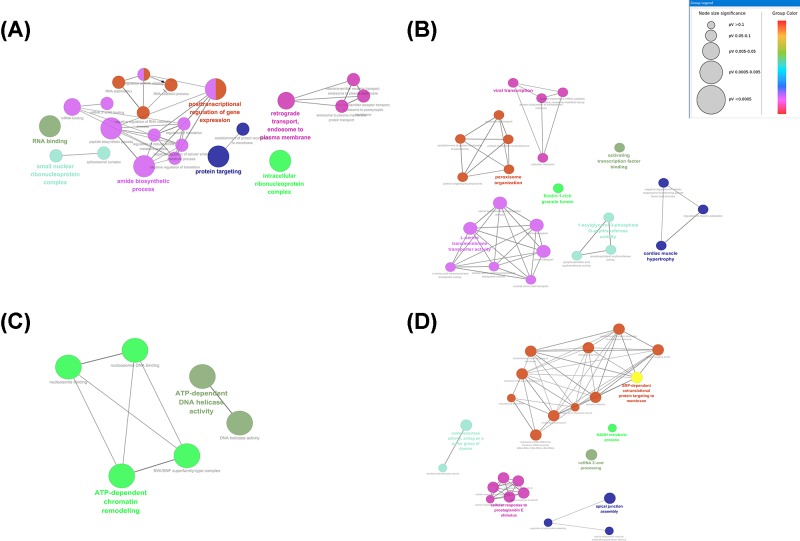
GO terms shows as an interaction network using Cytoscape plug-in ClueGO Plot **(A)** is for brown module, plot **(B)** is for the black module, plot **(C)** is for dark red module, and plot **(D)** is for midnight blue module.

**Figure 9:**
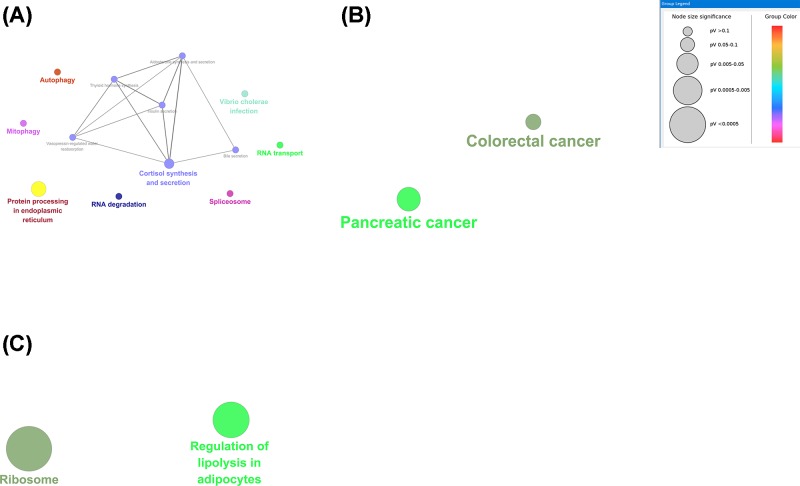
KEGG pathways displayed as an interaction network using Cytoscape plug-in ClueGO Plot **(A)** is for the brown module, plot **(B)** is for the black module, and plot **(C)** is for dark red module.

To explore the potential biological function of critical modules, we performed the functional enrichment analysis of GO and KEGG category. There was a significant difference in the biological processes that different modules were enriched in Figure 8 (Table 2). The results of GO analysis for brown module revealed that GO categories enriched in the biological process (*n=*25), cellular component (*n=*3), and molecular function (*n=*3). Genes in the brown module were largely enriched in GO:0034248 (regulation of cellular amide metabolic process), GO:0010608 (post-transcriptional regulation of gene expression), and GO:0043487 (regulation of RNA stability). The GO enrichment categories are exhibited in Figure 8A. A total of 15 KEGG pathways focussing on the biological pathways were enriched, including autophagy, spliceosome, and mitophagy. The KEGG pathway enrichment categories are exhibited in Figure 9A. Gene in the black module was mainly enriched in GO:0000184 (nuclear-transcribed mRNA catabolic process, nonsense-mediated decay), GO:0033613 (activating transcription factor binding), GO:0022626 (cytosolic ribosome) (Figure 8B). Two KEGG pathways focussing on the biological pathways were enriched in the black module, including colorectal cancer and pancreatic cancer (Figure 9B). Gene in the dark red module was mainly enriched in GO:0003678 (DNA helicase activity), GO:0004003 (ATP-dependent DNA helicase activity), and GO:0070603 (SWI/SNF superfamily-type complex) (Figure 8C). However, genes in dark red module did not significantly enrich any pathways. Genes in midnight blue module were enriched in GO:0043628 (ncRNA 3′-end processing), GO:0006734 (NADH metabolic process), and GO:0045047(protein targeting to ER) (Figure 8D). Genes in midnight blue module played critical roles in ribosome and regulation of lipolysis in adipocytes pathway (Figure 9C).

**Table 2:** The top five GO enrichment analysis of genes in the co-expression module

Module	Term	count	%	*P*-value
Brown	GO:0043487∼regulation of RNA stability	10	3.40	0.00
	GO:0034248∼regulation of cellular amide metabolic process	19	6.46	0.00
	GO:0010608∼posttranscriptional regulation of gene expression	25	8.50	0.00
	GO:0030529∼intracellular ribonucleoprotein complex	37	12.59	0.00
	GO:0043487∼regulation of RNA stability	10	3.40	0.00
Black	GO:0033613∼activating transcription factor binding	4	2.38	0.03
	GO:0022626∼cytosolic ribosome	6	3.57	0.00
	GO:0000184∼nuclear-transcribed mRNA catabolic process, nonsense-mediated decay	6	3.57	0.00
	GO:1904813∼ficolin-1-rich granule lumen	6	3.57	0.00
	GO:0019083∼viral transcription	8	4.76	0.00
Dark red	GO:0003678∼DNA helicase activity	3	6.82	0.00
	GO:0004003∼ATP-dependent DNA helicase activity	3	6.82	0.00
	GO:0031491∼nucleosome binding	3	6.82	0.00
	GO:0043044∼ATP-dependent chromatin remodeling	4	9.09	0.00
	GO:0070603∼SWI/SNF superfamily-type complex	4	9.09	0.00
Midnight blue	GO:0045047∼protein targeting to ER	5	5.88	0.00
	GO:0022626∼cytosolic ribosome	5	5.88	0.00
	GO:0042274∼ribosomal small subunit biogenesis	4	4.71	0.00
	GO:0006734∼NADH metabolic process	3	3.53	0.00
	GO:0043628∼ncRNA 3′-end processing	3	3.53	0.00

### Module visualization and hub genes

**Figure 10:**
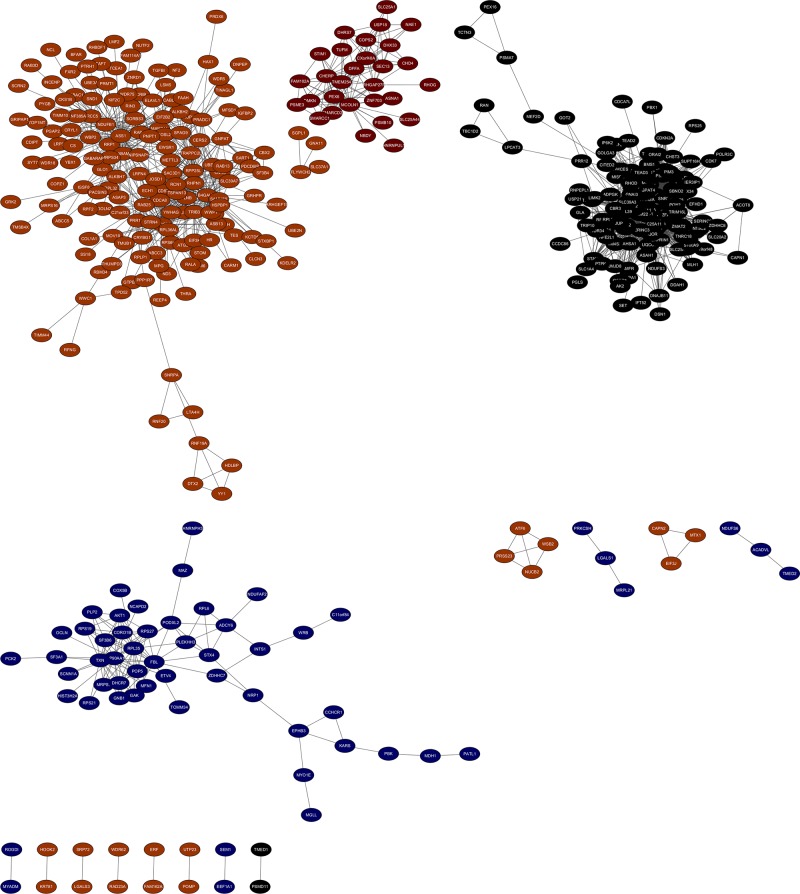
The visualization of four key modules Black nodes, black module; dark red nodes, dark red module; brown nodes, brown module; and midnight blue nodes, midnight blue module.

**Figure 11:**
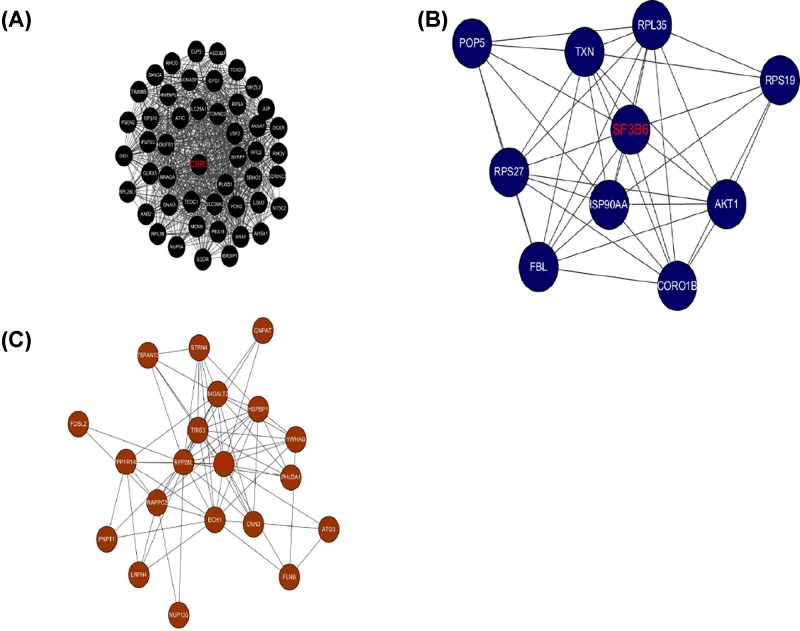
Three significant modules identified from the whole network using Cytoscape plug-in MCODE with a score of >6.0 Module I: black module, MCODE score = 34.979; module II: midnight blue module, MCODE score = 8.889; module III: brown module, MCODE score = 7.579. The hub genes in the modules were bold with red.

We chose gene pairs in the module which weight >0.05 and co-expression network was constructed using Cytoscape (Figure 10). The sub-network was also identified via Cytoscape plug-in MCODE. In total, three sub-co-expression networks (modules 1, 2, and 3) with score >6 were detected by MCODE (Figure 11). Each score was 34.979, 8.889, and 7.579, respectively. The three sub-co-expression networks belong to black module, midnightblue, and brown. Amongst these genes, CBR3, SF3B6, and RHPN1 were the hub gene in the sub-network.

### Validation of hub genes

**Figure 12:**
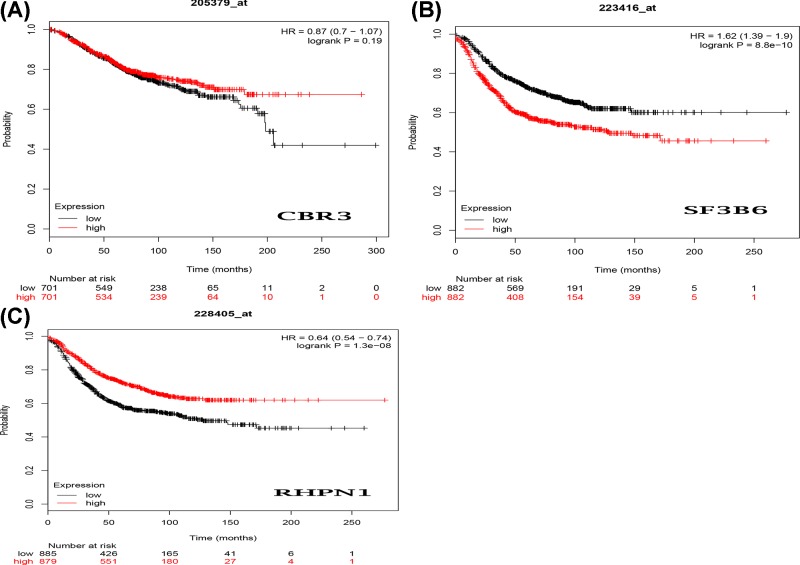
The prognostic value of mRNA level of hub genes in BC patients (OS in Kaplan–Meier plotter) **(A)** CBR3 (205379_at); **(B)** SF3B6 (223416_at); **(C)** RHPN1 (228405_at).

**Figure 13:**
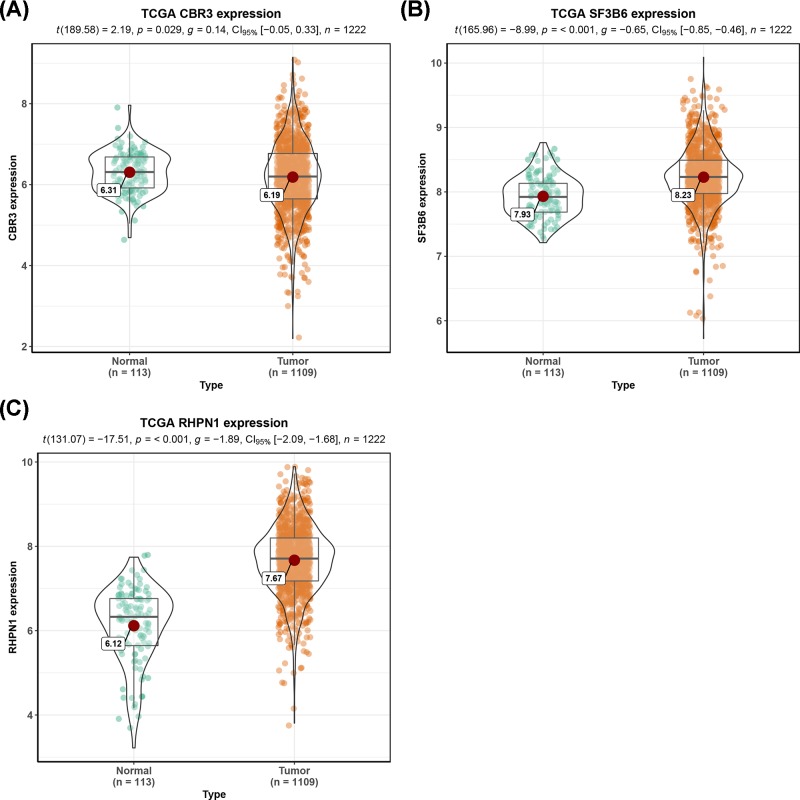
The mRNA expression level of three key genes between BRCA and adjunct noncancerous breast tissues based on TCGA data **(A)** CBR3; **(B)** SF3B6; **(C)** RHPN1.

**Figure 14:**
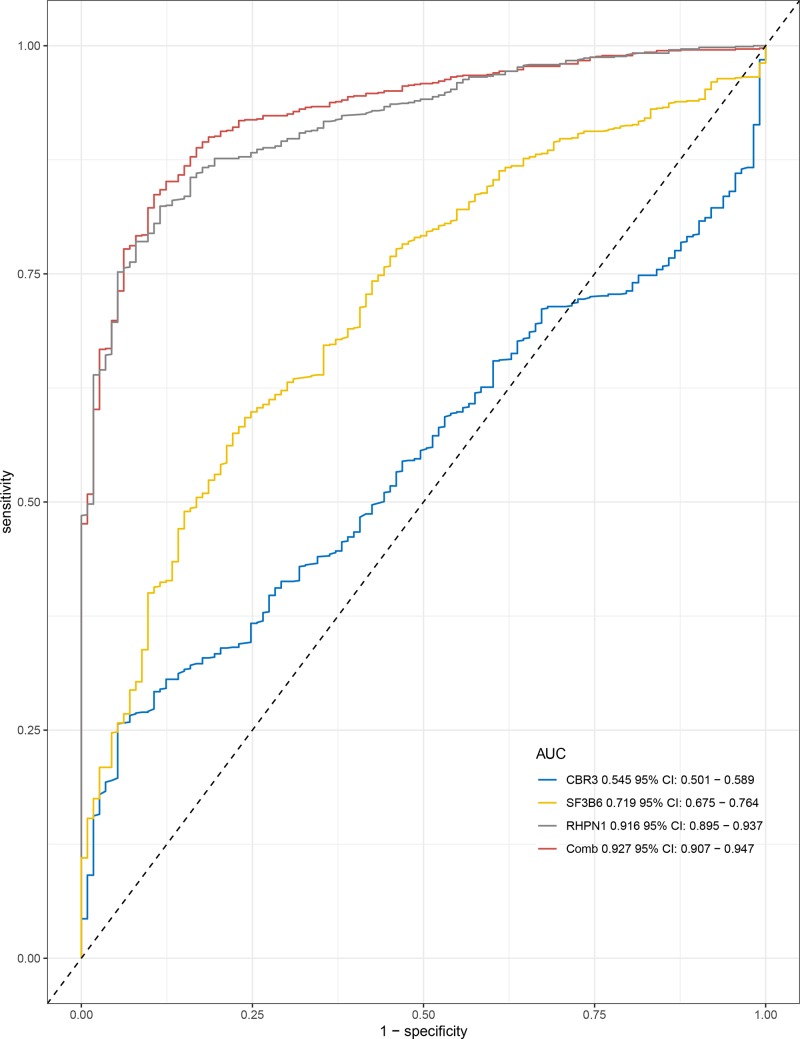
The ROC curves analysis for diagnostic power prediction by the three key genes

**Figure 15:**
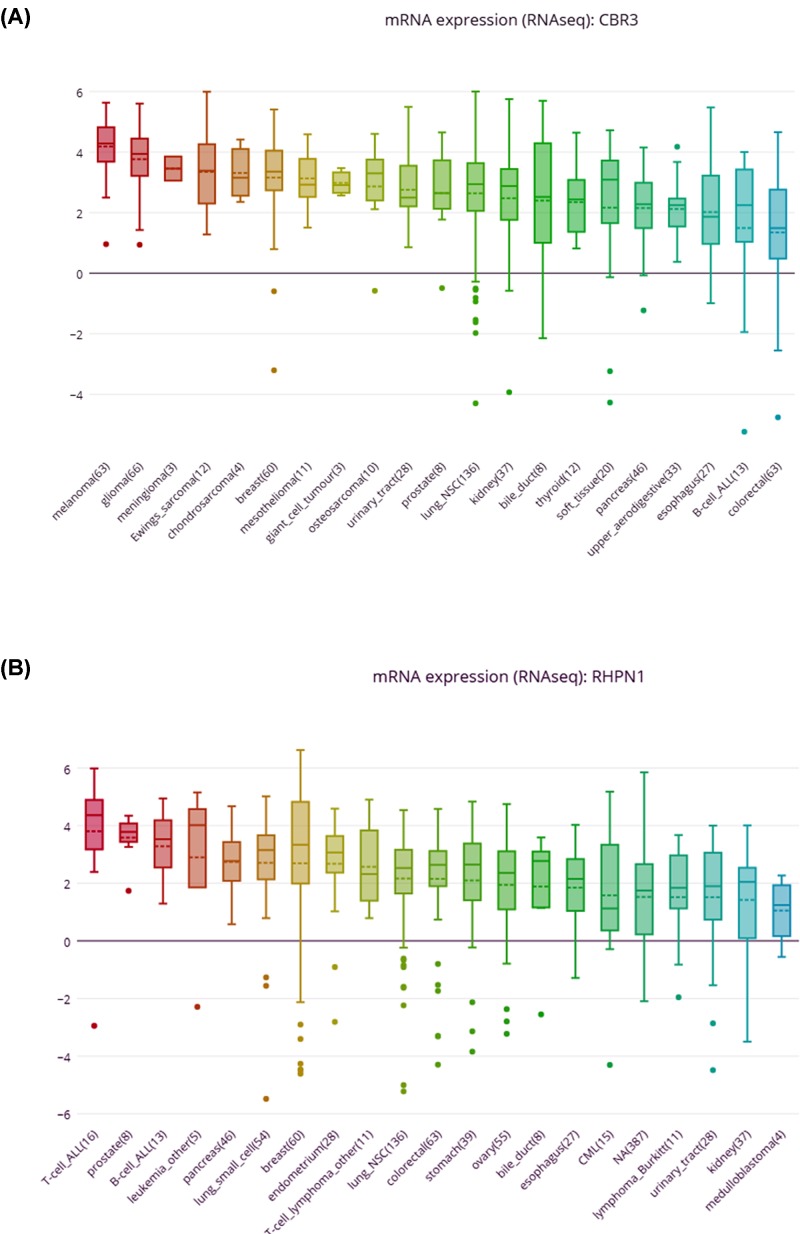
The two mRNA (CBR3 and RHPN1) were high expressed in kidney cancer cell lines from CCLE analysis

The Kaplan–Meier curve and log-rank test analyses revealed that the mRNA level of three hub genes was significantly associated with OS (*P*<0.05) (Figure 12) in all BC patients. The BC patients with higher mRNA levels of CBR3 and SF3B6 or lower mRNA level of RHPN1 were predicted to have a better OS. The three hub gene expression level was extracted in TCGA BRCA cohort. Compared with normal samples, the three hub gene expression level was higher in the tumor samples (Figure 13). We employed ROC curves to evaluate the prognostic power of three hub genes. The combined AUC for the three biomarkers prognostic model was 0.927 (0.907−0.947) (Figure 14). The CCLE database analysis demonstrated that the two hub gene expression level of CBR3 and RHPN1 ranked higher in a variety of cancer cell line (Figure 15).

## Discussion

BC is still one of the most common and malignant tumors by complex molecular and cellular heterogeneity [5]. This disease largely influences women in their 40–60s. It is the second most cancer now, just after lung cancer, the principal cause of death from cancer amongst women both in developing and developed countries [2,3]. BC cell lines may mirror many of the molecular characteristics of the tumors from which they were derived, and are therefore a useful preclinical model in which to explore strategies for predictive marker development. However, the mechanisms of critical pathways and their interactions involved in the occurrence and development of BC, remain largely unknown. Therefore, understanding of BC biology may provide clinicians with new tools that can be used for the treatment of this disease.

Many successful efforts were employed to examine the complex molecular and cellular nature of BC through basic genetic and molecular study, and various novel genes that are involved in BC have been identified [19–21]. Although individual gene or protein alone can produce an important role in the development and metastasis of BC, determining individual gene expression levels does not facilitate a comprehensive understanding of cancer cell development [22]. Due to rapid technological breakthroughs of genome-wide sequencing, the research of clinical issues and related pathological mechanisms in various cancers has been developed [23]. Weighted gene co-expression network analysis (WGCNA) is a method frequently used in the co-expression module correlation analysis by microarray samples. WGCNA is also a powerful tool to examine the potential gene correlation structures within the gene expression data. Therefore, the WGCNA method was employed to construct robust gene co-expression networks. These modules were established in terms of large-scale gene expression profiles and the distinction of centrally located genes (hub genes) that drive key cellular signaling pathways [24]. The WGCNA approach has provided functional interpretation tools in systems biology and led to new insights into the molecular and pathological mechanisms in several diseases [25,26]. There are no reports applying WGCNA to systematically identify gene co-expression networks associated with BC cell lines. To fulfill this gap, the WGCNA was applied to construct a co-expression network amongst each phenotype of BC cell line and calculated module-trait correlations based on one public microarray datasets (GSE78000), which included 36 samples and 22,615 genes. A total of 28 co-expression modules were constructed by WGCNA method, which helps clinicians to detect the association between BC cell line transcriptome and phenotype. We found four co-expression modules that relate to phenotype (basal, claudin-low, luminal, unknown, and non-malignant). Using the enrichment and functional analysis of Cytoscape plug-in ClueGO, we found that the KEGG pathway of targetted genes in the brown module was mainly involved in the pathway of the autophagy, spliceosome, and mitophagy, the black module was mainly enriched in the pathway of colorectal cancer and pancreatic cancer, and genes in midnightblue module played critical roles in ribosome and regulation of lipolysis in adipocytes pathway. Three hub genes CBR3, SF3B6, and RHPN1 may play an important role in the development and malignancy of BC. The genes stood out for their association with the diffuse histological subtype.

CBR3 (carbonyl reductase 3) is a protein-coding gene. Amongst its related pathways are metabolism and cytochrome P450 – arranged by substrate type. GO annotations related to this gene include oxidoreductase activity and carbonyl reductase (NADPH) activity. An important paralog of this gene is CBR1. At present, CBR3 has become potential biomarkers in uterine leiomyomas disease [27], type 2 diabetes [28], oral squamous cell carcinomas [29], and BC [30]. CBR3 mRNA expression could regulate human cancer cell lines by the Nrf2 pathway [31]. CBR3 is also proven a novel target gene of inflammatory stimuli [32]. Hertz et al. demonstrate that SNPs in CBR3 was correlated with chronic cardiotoxicity in a cohort of BC patients treated with anthracyclines [33].

SF3B6 (splicing factor 3b subunit 6) is a protein-coding gene. Amongst its related pathways are gene expression and mRNA splicing - major pathway. GO annotations related to this gene include nucleic acid binding and nucleotide binding. Siebring-van et al. identified that silencing SF3A3 yielded much stronger cytotoxicity to non-small cell lung cancer cells than to lung fibroblasts, suggesting that the gene could represent useful therapeutic targets [34].

RHPN1 (Rhophilin Rho GTPase binding protein 1) is a protein-coding gene. Amongst its related pathways are ectoderm differentiation and RHO GTPases activate rhotekin and rhophilins. An important paralog of this gene is RHPN2. Lal MA reported that Rhophilin-1 is a key regulator of the podocyte cytoskeleton, and is essential for glomerular filtration [35].

In this work, our findings demonstrated that black module and brown module, midnight blue module, and dark red module were regarded as the most critical module in the development and pathological phenotype of BC. And the hub gene CBR3, SF3B6, and RHPN1 were found to be significantly in three modules, which may play as the potential diagnostic and prognostic biomarkers of BC, However, the biological function of these three genes needs to be further validated with more experiment.

## Availability of data and material

Data availability could be obtained from TCGA website.

## Supporting information

**Supplementary Table S1:** The genes of four modules

## Abbreviations

CBR3: carbonyl reductase 3
GEO: gene expression omnibus
GO: gene ontology
GS: gene significance
KEGG: Kyoto Encyclopedia of Genes and Genomes
MCODE: molecular complex detection
ME: module eigengene
MM: module membership
ROC: receiver operating characteristic
TOM: topological overlap matrix

## References

[1] Jemal A., Bray F., Center M.M., Ferlay J., Ward E. and Forman D. (2011) Global cancer statistics. CA Cancer J. Clin. 61, 69–90 10.3322/caac.20107 21296855

[2] McGuire S. (2016) World Cancer Report 2014. Geneva, Switzerland: World Health Organization, International Agency for Research on Cancer, WHO Press, 2015. Ad. Nutr. 7, 418–419, PubMed Central PMCID: PMC4785485. 10.3945/an.116.012211 26980827 PMC4785485

[3] Siegel R.L., Miller K.D. and Jemal A. (2017) Cancer statistics, 2017. CA Cancer J. Clin. 67, 7–30 10.3322/caac.21387 28055103

[4] Reis-Filho J.S. and Pusztai L. (2011) Gene expression profiling in breast cancer: classification, prognostication, and prediction. Lancet 378, 1812–1823 10.1016/S0140-6736(11)61539-0 22098854

[5] Marusyk A. and Polyak K. (2010) Tumor heterogeneity: causes and consequences. Biochim. Biophys. Acta 1805, 105–117, PubMed Central PMCID: PMC2814927 19931353 10.1016/j.bbcan.2009.11.002 PMC2814927

[6] Zhang B. and Horvath S. (2005) A general framework for weighted gene co-expression network analysis. Stat. Appl. Genet. Mol. Biol. 4, Article17 10.2202/1544-6115.1128 16646834

[7] Langfelder P. and Horvath S. (2008) WGCNA: an R package for weighted correlation network analysis. BMC Bioinformatics 9, 559, PubMed Central PMCID: PMC2631488 10.1186/1471-2105-9-559 19114008 PMC2631488

[8] Tang Y., Ke Z.P., Peng Y.G. and Cai P.T. (2018) Co-expression analysis reveals key gene modules and pathway of human coronary heart disease. J. Cell. Biochem. 119, 2102–2109 10.1002/jcb.26372 28857241

[9] Wan Q., Tang J., Han Y. and Wang D. (2018) Co-expression modules construction by WGCNA and identify potential prognostic markers of uveal melanoma. Exp. Eye Res. 166, 13–20 10.1016/j.exer.2017.10.007 29031853

[10] Yepes S., Lopez R., Andrade R.E., Rodriguez-Urrego P.A., Lopez-Kleine L. and Torres M.M. (2016) Co-expressed miRNAs in gastric adenocarcinoma. Genomics 108, 93–101 10.1016/j.ygeno.2016.07.002 27422560

[11] Huang H., Zhang Q., Ye C., Lv J.M., Liu X., Chen L. (2017) Identification of prognostic markers of high grade prostate cancer through an integrated bioinformatics approach. J. Cancer Res. Clin. Oncol. 143, 2571–2579 10.1007/s00432-017-2497-0 28849390 PMC11818995

[12] Guo Y. and Xing Y. (2016) Weighted gene co-expression network analysis of pneumocytes under exposure to a carcinogenic dose of chloroprene. Life Sci. 151, 339–347 10.1016/j.lfs.2016.02.074 26916823

[13] Daemen A., Griffith O.L., Heiser L.M., Wang N.J., Enache O.M., Sanborn Z. (2013) Modeling precision treatment of breast cancer. Genome Biol. 14, R110, PubMed Central PMCID: PMC3937590 10.1186/gb-2013-14-10-r110 24176112 PMC3937590

[14] Langfelder P. and Horvath S. (2012) Fast R functions for robust correlations and hierarchical clustering. J. Stat. Softw. 46, PubMed PMID: 23050260; PubMed Central PMCID: PMC3465711 10.18637/jss.v046.i11 23050260 PMC3465711

[15] Bindea G., Mlecnik B., Hackl H., Charoentong P., Tosolini M., Kirilovsky A. (2009) ClueGO: a Cytoscape plug-in to decipher functionally grouped gene ontology and pathway annotation networks. Bioinformatics 25, 1091–1093, PubMed Central PMCID: PMC2666812 10.1093/bioinformatics/btp101 19237447 PMC2666812

[16] Gyorffy B., Surowiak P., Budczies J. and Lanczky A. (2013) Online survival analysis software to assess the prognostic value of biomarkers using transcriptomic data in non-small-cell lung cancer. PLoS ONE 8, e82241, PubMed Central PMCID: PMC3867325 10.1371/journal.pone.0082241 24367507 PMC3867325

[17] Gyorffy B., Lanczky A., Eklund A.C., Denkert C., Budczies J., Li Q. (2010) An online survival analysis tool to rapidly assess the effect of 22,277 genes on breast cancer prognosis using microarray data of 1809 patients. Breast Cancer Res. Treat. 123, 725–731 10.1007/s10549-009-0674-9 20020197

[18] Barretina J., Caponigro G., Stransky N., Venkatesan K., Margolin A.A., Kim S. (2012) The Cancer Cell Line Encyclopedia enables predictive modelling of anticancer drug sensitivity. Nature 483, 603–607, PubMed Central PMCID: PMC3320027 10.1038/nature11003 22460905 PMC3320027

[19] Ma X.J., Wang Z., Ryan P.D., Isakoff S.J., Barmettler A., Fuller A. (2004) A two-gene expression ratio predicts clinical outcome in breast cancer patients treated with tamoxifen. Cancer Cell 5, 607–616 10.1016/j.ccr.2004.05.015 15193263

[20] Terunuma A., Putluri N., Mishra P., Mathe E.A., Dorsey T.H., Yi M. (2014) MYC-driven accumulation of 2-hydroxyglutarate is associated with breast cancer prognosis. J. Clin. Invest. 124, 398–412, PubMed Central PMCID: PMC3871244 10.1172/JCI71180 24316975 PMC3871244

[21] Yao D.J., Qiao S., Zhang Y., Zhao Y.T. and Yuan C.H. (2017) Correlation between expression of LRP16, Ki67 and EGFR and breast cancer clinical pathologic factors and prognosis. Eur. Rev. Med. Pharmacol. Sci. 21, 47–51, PubMed PMID: 28745792 28745792

[22] Wang X., Qian H. and Zhang S. (2014) Discovery of significant pathways in breast cancer metastasis via module extraction and comparison. IET Syst. Biol. 8, 47–55 10.1049/iet-syb.2013.0041 25014225 PMC8687293

[23] Stratton M.R., Campbell P.J. and Futreal P.A. (2009) The cancer genome. Nature 458, 719–724, PubMed Central PMCID: PMC2821689 10.1038/nature07943 19360079 PMC2821689

[24] Miller J.A., Horvath S. and Geschwind D.H. (2010) Divergence of human and mouse brain transcriptome highlights Alzheimer disease pathways. Proc. Natl Acad. Sci. U.S.A 107, 12698–12703, PubMed Central PMCID: PMC2906579 10.1073/pnas.0914257107 20616000 PMC2906579

[25] Horvath S. and Dong J. (2008) Geometric interpretation of gene coexpression network analysis. PLoS Comput. Biol. 4, e1000117, PubMed Central PMCID: PMC2446438 10.1371/journal.pcbi.1000117 18704157 PMC2446438

[26] Levine A.J., Miller J.A., Shapshak P., Gelman B., Singer E.J., Hinkin C.H. (2013) Systems analysis of human brain gene expression: mechanisms for HIV-associated neurocognitive impairment and common pathways with Alzheimer’s disease. BMC Med. Genet. 6, 4, PubMed Central PMCID: PMC3626801 10.1186/1755-8794-6-4 PMC3626801 23406646

[27] Liu X., Liu Y., Zhao J. and Liu Y. (2018) Screening of potential biomarkers in uterine leiomyomas disease via gene expression profiling analysis. Mol. Med. Rep. 17, 6985–6996 29568968 10.3892/mmr.2018.8756 PMC5928645

[28] Chang Y.C., Liu P.H., Tsai Y.C., Chiu Y.F., Shih S.R., Ho L.T. (2012) Genetic variation in the carbonyl reductase 3 gene confers risk of type 2 diabetes and insulin resistance: a potential regulator of adipogenesis. J. Mol. Med. 90, 847–858 10.1007/s00109-012-0898-8 22527884

[29] Ohkura-Hada S., Kondoh N., Hada A., Arai M., Yamazaki Y., Shindoh M. (2008) Carbonyl reductase 3 (CBR3) mediates 9-cis-retinoic acid-induced cytostatis and is a potential prognostic marker for oral malignancy. Open Dent. J. 2, 78–88, PubMed Central PMCID: PMC2581532 10.2174/1874210600802010078 19088887 PMC2581532

[30] Lal S., Sandanaraj E., Wong Z.W., Ang P.C., Wong N.S., Lee E.J. (2008) CBR1 and CBR3 pharmacogenetics and their influence on doxorubicin disposition in Asian breast cancer patients. Cancer Sci. 99, 2045–2054 10.1111/j.1349-7006.2008.00744.x 19016765 PMC11160041

[31] Ebert B., Kisiela M., Malatkova P., El-Hawari Y. and Maser E. (2010) Regulation of human carbonyl reductase 3 (CBR3; SDR21C2) expression by Nrf2 in cultured cancer cells. Biochemistry 49, 8499–8511 10.1021/bi100814d 20806931

[32] Malatkova P., Ebert B., Wsol V. and Maser E. (2012) Expression of human carbonyl reductase 3 (CBR3; SDR21C2) is inducible by pro-inflammatory stimuli. Biochem. Biophys. Res. Commun. 420, 368–373 10.1016/j.bbrc.2012.03.002 22425771

[33] Hertz D.L., Caram M.V., Kidwell K.M., Thibert J.N., Gersch C., Seewald N.J. (2016) Evidence for association of SNPs in ABCB1 and CBR3, but not RAC2, NCF4, SLC28A3 or TOP2B, with chronic cardiotoxicity in a cohort of breast cancer patients treated with anthracyclines. Pharmacogenomics 17, 231–240, PubMed Central PMCID: PMC5558515 10.2217/pgs.15.162 26799497 PMC5558515

[34] Siebring-van Olst E., Blijlevens M., de Menezes R.X., van der Meulen-Muileman I.H., Smit E.F. and van Beusechem V.W. (2017) A genome-wide siRNA screen for regulators of tumor suppressor p53 activity in human non-small cell lung cancer cells identifies components of the RNA splicing machinery as targets for anticancer treatment. Mol. Oncol. 11, 534–551, PubMed Central PMCID: PMC5527466 10.1002/1878-0261.12052 28296343 PMC5527466

[35] Lal M.A., Andersson A.C., Katayama K., Xiao Z., Nukui M., Hultenby K. (2015) Rhophilin-1 is a key regulator of the podocyte cytoskeleton and is essential for glomerular filtration. J. Am. Soc. Nephrol. 26, 647–662, PubMed Central PMCID: PMC4341472 10.1681/ASN.2013111195 25071083 PMC4341472

