# Supplementary material for: Co-expression of key gene modules and pathways of human breast cancer cell lines
Source: Biosci Rep. 2019 Jul 19;39(7):BSR20181925. doi: 10.1042/BSR20181925 (PMC6639467; doi:10.1042/BSR20181925)
Supplement: Supplementary file 1 [file bsr20181925_Supp1.pdf]

Supplement Table 1: The genes of four modules.

Module	Gene
Black	RPL39;RPL13;RPS10;IFITM3;RPS25;RPSA;ARPC1B;PUF60;S100A9;DCXR;PSMA7;SERINC2;JUP;CAPN1;SET;SLC25A39;RAN;ARF5;TM4SF1;FKBP8;C19orf48;RHOD;TIMM8B;WDR34;GOT2;AHSA1;HNRNPL;TUBA4A;PIGT;LSM3;PSMD11;EFHD1;PBX1;UQCC3;AIP;FTSJ3;USF2;CAPZA1;RRM2;SQOR;NDUFB11;ATIC;ZMAT2;PLOD1;TRIP10;ADAR;ANXA7;NDUFS3;PIM3;WFS1;NFE2L1;SLC25A11;SNX17;ACAT2;XAB2;SNRPF;SUPT16HMISP;PGLS;MBOAT7;TOMM22;PMEPA1;NOTCH3;JMJD8;SERINC3;RPL26L1;CLN6;RRAGA;AMFR;RFC2;PIN1;DEK;CCDC86;GPAT4;HADHB;ARAF;DNAJB11;RHOV;CAPRIN1;LIMK2;CITED2;GORASP2;MRPL40;SLC38A1;ZNF217;PPL;RNPEPL1;PTRHD1;STAU1;AK2;MCM6;SBNO2;ACOT8;CDKN2A;OSER1;GYS1;ASAH1;HMCES;ULK1;TNRC18;SLC39A3;PELP1;GYG1;LPCAT3;POLRMT;COPRS;PON2;GNAI3;ZFAND6;SLC20A2;TEAD2;SLC1A4;POLR3C;TMED1;TEDC1;HDGFL2;CEP55;TRIM16L;GOLGA3;GLRX3;PEX16;BMS1;CBR3;SMAD3;ZDHHC8;HSD3B7;FAM46B;DHX34;TEAD3;PEX19;C11orf52;PTPN6;CMPK1;GMPR2;PSEN2;TBC1D2;CDCA7L;TMEM223;MEF2D;PIR;PRRC1;NR2F2;BTBD10;HOXC13;MLH1;ELP5;PCED1A;MED25;IFT52;NUP54;USP21;PRR14;IER3IP1;MYO9B;DDAH1;ORAI2;IER5L;CHST3;DSN1;TCTN3;NT5C2;MPZL2;IP6K2;CDK7;ADPGK;PRR12;GLA
Brown	RPLP1;ND3;UBB;TMSB4X;YBX1;NCL;PRMT1;CAPN2;RPL36AL;MYL12B;ECH1;ASS1;EDF1;PEBP1;EIF3K;KRT81;CCT5;RAB25;BTF3;NUTF2;PPP1R14B;IFITM2;LGALS3;MRPS34;PSMB7;SLC9A3R2;RABAC1;TSPAN15;HNRNPAB;ATF4;CKS1B;TRAPPC5;CERS2;GABARAP;FLNB;ARPC3;PYGB;TSPAN13;YIF1A;HAX1;PRDX6;CD82;MARS;HNRNPC;XRCC5;RAD23A;CSE1L;GRK2;TRAPPC2L;ATP5F1;PRNP;SF3B4;CNN3;WBP2;TRIB3;CS;TIMM10;IGFBP2;CLUH;KDELR2;RAB13;SND1;PDIA6;SLC39A7;GLO1;RAB3D;GCN1;NDUFB1;COPZ1;NDUFAB1;KHSRP;YWHAG;HDLBP;WDR18;DKC1;PRR15L;AGTRAP;HSPBP1;C21orf33;RAB11A;PACSIN3;STRN4;NIPSNAP1;EWSR1;SART1;SCRIB;SNRPA;ASPH;HIST1H4C;LDLR;DNMT1;RCN1;GRHPR;UBE2E1;REEP4;GNA11;TINAGL1;FAM162A;ABCC3;WSB2;POMP;IMPA2;KIF2C;LMF2;CARM1;IGSF8;SORD;ATP6V0E2;MICALL1;LRFN4;RHBDF1;TIMM17A;SYT7;STOM;MPG;SRP72;WWC1;ZNF385A;CDCA8;RPL39L;TIMM44;LRP3;PRSS23;FHOD1;FLYWCH2;TOP1MT;C1orf35;INCENP;SDCCAG3;METTL3;TCEA1;B4GALT2;RPS6KA1;GTPBP2;CTSF;BRD2;TMUB1;ADCY3;MRPL32;EVPL;PIP5K1A;SORBS3;MANBAL;MRPS16;TAF7;EIF2B2;PSKH1;CBX2;RRP1;LRRC45;TM7SF3;TES;EIF2S1;LTA4H;KCTD5;DNPEP;ZNRD1;TGFBI;LSM12;WDR5;ERF;ALKBH7;CCDC167;CDIPT;JOSD1;BRD4;EIF3J;MGP;RHPN1;HOOK2;IMMT;SS18;THRAP3;SAMM50;TLCD1;CRB3;NUCB2;SAT2;ATP13A3;RFNG;SPOUT1;SSR1;MTX1;MFSD1;XPO5;GAL;FOSL2;PRADC1;F3;TMPRSS13;RPP25L;ZNF687;CREB3;HECTD3;FAAH;SAR1A;SMG9;UBE2N;MYORG;RAB20;YY1;PGAP2;TMEM30A;ACBD3;RALA;SLC37A1;CMTM6;WDR62;SKIV2L2;PDCD6IP;DTX2;CCDC92;ALKBH2;GCLC;RBM34;CENPF;MRPS30;CRYBG1;RPF2;DALRD3;GNPAT;SPAG9;WDR75;ELOA;ASXL1;MOV10;PPP1R7;RNF20;TPD52;ATF6;SIPA1L3;FXR2;MRPS27;STXBP1;GRAMD1A;HR;RNF220;SCRN2;ZMPSTE24;PPP1R18;TMEM160;IGBP1;TGOLN2;DNAJC11;ADAM10;ZDHHC3;SAC3D1;PNPT1;NF2;THRA;TARBP2;IGSF9;PHLDA1;PTRH1;TAOK2;ENO2;COL1A1;SLU7;LSM5;CCDC97;KBTBD2;ASAP3;TCEA2;PFKFB3;MAN2B2;UTP23;COL6A1;NUP133;DHX36;CABLES1;ATG3;SGPL1;WWP1;ABCC5;SMARCD1;ELAVL1;POGZ;RI

	N3;CLCN3;ASB13;FAM114A1;BFAR;CRYL1;DHX8;THUMPD3;TRIM56;COA6;HIRIP3;GRIPAP1;UBE3A;PIDD1;BABAM2;ARHGEF19;RNF19A
Darkred	TUFM;SLC25A1;SMARCD2;TFDP1;RUVBL1;ECI1;HNRNPUL1;PSME3;MRPL14;FAM102A;ASNA1;CHD4;PSMB10;RHOG;C11orf58;SEC13;DHRS7;NBDY;COPS2;SLC20A1;PABPN1;KAT2A;CHERP;LRWD1;VAC14;STIM1;ELF4;NAE1;SMARCC1;PEX6;TMEM254;KATNB1;MCOLN1;G3BP1;SLC25A44;ZNF703;USP18;DMKN;CXorf40A;DFFA;DHX33;EPS8;ARHGAP27P1
Midnightblue	LGALS1;RPL35;EEF1A1;RPL6;RPS19;RPS27;RPS21;HSP90AA1;TPI1;TXN;FBL;COX5B;PLP2;MAZ;SEM1;MYL12A;CORO1B;NDUFS6;DHCR7;AKT1;PRKCSH;TMED2;ACADVL;SCNN1A;RHOC;GNB1;MDH1;KARS;EMC10;CUTA;GSTK1;INTS1;MRPL21;EHD1;MYADM;NCAPD2;PCK2;MGLL;SF3B6;HNRNPH3;DNAJB1;IRX3;GTPBP4;TOMM34;HIST3H2A;SNW1;ROGDI;PODXL2;POP5;CDK10;ADCY6;GAK;ZDHHC7;EPHB3;PSMG2;ZC3H18;NDUFAF2;PATL1;STX4;ETV4;MAP7;SF3A1;CAMSAP3;SERTAD1;PNISR;SLC4A11;SP3;PLEKHH3;SRCAP;ZNF32;MFN1;PBK;TXNDC17;OCLN;UBFD1;C11orf84;MRPS9;WRB;ABRACL;CCHCR1;ZNF213;NRP1;MYO1E;DTX3
